# Determinants of measles second dose vaccination dropout among children aged 18–24 months in Ejere woreda, central Ethiopia; unmatched case-control study

**DOI:** 10.3389/fped.2024.1432762

**Published:** 2024-09-18

**Authors:** Kitessa Nurgi, Seifadin Ahmed, Gemechu Ganfure, Gemechu Gelan Bekele

**Affiliations:** ^1^ Department of Public Health, Ejere Woreda Health Office, West Shewa, Ethiopia; ^2^ Department of Public Health, College of Medicine and Health Science, Arsi University, Asella, Ethiopia; ^3^ Department of Pediatrics and Child Health, College of Health Sciences and Referral Hospital, Ambo University, Ambo, Ethiopia; ^4^ Department of Midwifery, College of Health Sciences, Mada Walabu University, Shashamene, Ethiopia

**Keywords:** measles, dropout, determinants, Ethiopia, vaccine

## Abstract

**Background:**

Measles continues to pose a significant public health challenge, especially in low- and middle-income countries. Despite the implementation of national vaccination programs, measles outbreaks persist in some parts of Ethiopia, and the determinants of dropout from the second measles vaccine dose are not well understood. Hence, this study aimed to assess determinants of measles second dose vaccination dropout among children aged 18–24 months in Ejere woreda, central Ethiopia.

**Methods:**

A community-based unmatched case-control design was conducted in the Ejere Woreda of the Oromia regional state in Ethiopia between February 14 and April 6, 2023. Data were collected using a pre-tested structured questionnaire. The collected data were coded and entered into Epi-data version 3.1 and then transported to SPSS version 27 for statistical analysis. Descriptive analysis like frequency, mean, and percentage was calculated. Binary and multivariable logistic regression analysis was done. Finally, variables with a *p*-value <0.05 were considered statistically significant.

**Result:**

A total of 446 mothers/caregivers, comprising 110 cases and 336 controls, participated in this study, making the response rate 97.8%. Lack of a reminder for the measles vaccine during postnatal care (PNC) (AOR = 5.19; 95% CI: 2.34, 7.83), having ≤2 antenatal care (ANC) contacts (AOR = 4.95; 95% CI: 2.86, 9.24), long waiting times during previous vaccination (AOR = 2.78; 95% CI: 1.19, 4.38), children of mothers/caregivers without formal education (AOR = 6.46; 95% CI: 2.81, 11.71), mothers/caregivers of children who were unaware of the importance of the second dose of measles (AOR = 8.37; 95% CI: 4.22, 15.08), and mothers/caregivers whose children did not receive at least two doses of vitamin A (AOR = 4.05; 95% CI: 2.15, 8.11) were significant determinants of measles second dose vaccination dropout.

**Conclusion:**

Implementing targeted interventions during antenatal care and when mothers visit health facilities for other vaccines can significantly improve the uptake of the second dose of the measles vaccine. These strategies not only enhance overall vaccination coverage but also mitigate the risk of measles outbreaks in the community.

## Background

Measles is an acute, highly infectious, and potentially life-threatening disease caused by the morbillivirus, characterized by fever, rash, and respiratory symptoms. The disease is primarily transmitted through aerosolized droplets or direct contact with the secretions of infected individuals (1, 2).

Measles is almost entirely preventable through vaccination, with the World Health Organization (WHO) recommending two doses: the first at 9 months and the second between 15 and 18 months (3–5). While the first dose offers substantial protection against measles, the second dose is essential for developing robust and long-lasting immunity. By significantly boosting the immune response, the second dose protects over 95% of vaccinated individuals against the disease, thereby reducing the likelihood of outbreaks (5). Despite the availability of safe vaccines, approximately 136,000 measles-related deaths occurred globally in 2022, primarily among unvaccinated or under-vaccinated children (4, 5).

In 2021, the global coverage for the first dose of the measles-containing vaccine (MCV1) was 81%, while the coverage for the second dose (MCV2) was 71% (5, 6). In Africa, however, the rate of MCV1 was only 68%, and the coverage for MCV2 was at a mere 41%, resulting in a 27% default rate (4). This coverage remains below the 95% threshold required for two doses that are essential to protect communities from outbreaks (5).

In Ethiopia, the coverage of measles vaccination is concerning, with MCV1 only at 59% (7). The MCV2 program, introduced in 2018 and integrated into routine immunization in 2019, aims to enhance immunity and is administered at fifteen months of age (8). However, MCV2 coverage remains low, with only 12.36% reported in 2019 and 42.5% in 2022, indicating a significant dropout rate (9, 10).

Although measles can be prevented through vaccination, can lead to severe serious illness and long-term complications, including pneumonia, diarrhea, otitis media, encephalitis, subacute sclerosing panencephalitis, and death (11, 12).

Childhood vaccination dropout is influenced by various factors, including the mother's knowledge, age, marital status, educational level, socioeconomic status, antenatal care attendance, birth order, postnatal care follow-up, understanding vaccination schedule, uptake of pentavalent 3 vaccines, Vitamin A vaccination status, and waiting time (13–18).

To eliminate measles by 2020, countries in the WHO African Region and partners aimed to achieve a coverage rate of at least 95% for MCV2 (1). Ethiopia has also developed a measles elimination strategy targeting the same goal (7, 11). However, a decline in vaccination coverage and an increase in dropout rate from vaccination resulted in significant and disruptive measles outbreaks in 22 countries in 2021, including Ethiopia (4).

There is limited evidence regarding the determinants of MCV2 dropout in Ethiopia. Moreover, previous studies on vaccination dropout in Ethiopia have examined all vaccinations collectively without specifically studying the determinants for measles vaccination dropout. This generalized approach may overlook the unique factors associated with measles vaccinations. By focusing solely on measles vaccination dropout, this study addresses this gap by providing a more nuanced understanding of the determinants specific to measles vaccination. Therefore, this study aimed to assess the determinants of measles second dose vaccination dropout among children aged 18–24 months in Ejere woreda, central Ethiopia, in 2023.

## Methods

### Study design, area and period

This community-based case-control study was conducted from February 14 to April 6, 2023. The choice of an unmatched case-control approach stemmed from the practical challenges of identifying suitable matches for all cases, considering the demographic diversity and logistical constraints of the study. The study was conducted in Ejere woreda (the third-level administrative division in Ethiopia, positioned below zones and regional states). Ejere is located in the West Shewa zone of the Oromia regional state in central Ethiopia. Ejere is one of the 22 woredas in the West Shewa zone, located 71 kilometers from Ambo, the zone's capital, and 43 kilometers west of, the country's capital. The Ejere woreda comprises three urban kebeles (the smallest administrative units responsible for local governance in Ethiopia) and 26 rural kebeles, with a total population of 132,489. This includes 27,955 individuals in the reproductive age group and 23,848 children under the age of five. The woreda is served by four public health centers, 26 rural health posts, and two private medical facilities.

### Population

The source population for this study consisted of mothers or caregivers residing in Ejere woreda with children aged between 18 and 24 months. Mothers or caregivers who had initiated the first dose of the measles vaccine for their children aged 18–24 months but had defaulted on the subsequent doses were included in the study as the study population for the case group. On the other hand, mothers or caregivers with children aged 18–24 months who had received both doses of the measles vaccine as per the routine schedule were included as the study population for the control group. However, mothers or caregivers who were seriously ill or had received the second dose of the measles vaccine through a campaign were excluded from the study.

### Sample size determination and sampling procedure

To calculate the sample size, the study used EPI Info software. It considered a 95% confidence level, 80% power, a design effect of 1.5, and a case-to-control ratio of 1:3. The study used penta-3 as a risk factor with an odds ratio of 3.45, with a 3.92% proportion of controls exposed to the second dose of measles (19). After accounting for a 10% non-response rate, the final estimated sample size became 456, consisting of 114 cases and 342 controls.

A multistage sampling approach was employed to select participants, including both cases and controls. The Ejere woreda was initially divided into urban and rural kebeles for stratification, from which eight rural kebeles and one urban kebele were randomly selected. A baseline survey was conducted before data collection to determine the measles vaccination status of children. Each eligible caregiver received a family folder labeled with their status, and a house code was assigned and recorded. The sample size was distributed proportionally among the kebeles based on population size. Finally, 114 cases and 342 controls were randomly selected from the baseline survey using a simple random technique for each group.

### Variables

#### Dependent variable

Defaulting from the second dose of measles vaccination.

#### Independent variables

Includes a range of factors that influence childhood immunization coverage, encompassing sociodemographic elements (e.g., maternal/caregiver education, residence, age, marital status, family size, occupation), child characteristics (age, birth order, sex), maternal/caregiver awareness, perceptions, and immunization history, as well as healthcare-related variables (ANC, place of delivery, PNC) and health system factors (travel time, HEW visits, vaccination waiting time, vaccination days per week, and Vitamin A vaccination status).

### Operational definition

#### Measles dropout

Children aged 15–18 months who have not received the MCV2 vaccine or have only received the first dose are classified as defaulters or dropped out of MCV2 (16).

#### Awareness of the schedule of the second dose of measles

This was assessed by asking four questions, including the age at which the measles vaccine is administered and the total number of doses needed for complete immunization. Each correct answer was assigned score of 1, while incorrect responses received a score of 0. The mean scores of participants were calculated, with those scoring above the mean were categorized as having good awareness, and those scoring below the mean were categorized as having poor awareness (20).

#### Perception towards benefits of the measles vaccine

A questionnaire with six questions was used to measure participant's opinions about their perception toward the benefits of the measles vaccine. The responses were given on a five-point Likert scale, ranging from strongly agree to strongly disagree. Strongly agree received the highest rating, while strongly disagree was assigned the lowest score. Subsequently, the average score for each concept was categorized into positive perceptions for scores above the mean and negative perceptions for scores below the mean (20).

### Data collection tools and techniques

A questionnaire was adapted from different pieces of literature (14, 16, 18, 21). It was designed to obtain formation from participants regarding their socio-demographics, maternal health care, health facilities, awareness of MCV2, perceptions toward the benefit of MCV2, and concurrently administered immunizations. The questionnaire was initially developed in English, and then translated into the local language, Afaan Oromo, and subsequently translated back into English to ensure consistency (Supplementary Table S1). Six nurse data collectors were trained to gather the data, while two senior public health officers supervised the data collection process.

### Data quality control

Data quality assurance was carried out in three stages: before, during, and after the data collection period. The investigator conducted a two-day training session for data collectors and supervisors, covering all six sections of the questionnaire. A pretest of the questionnaire was conducted on 5% of the sample size (including caregivers of 6 cases and 17 controls) in a non-selected kebele in Ejere woreda to ensure data reliability. Adjustments were made to certain questionnaire items based on the pretest results. Close and timely supervision was implemented throughout the data collection process to maintain the data quality.

### Data processing and analysis

The data were checked, coded, and entered into Epi-data 3.1, then exported to SPSS 27 for cleaning and analysis. The data was checked for missed values and outliers, and descriptive statistics were calculated. Multicollinearity between independent variables was assessed using variance inflation factors. The Hosmer-Lemeshow test was used to assess model fit, with the final model fitting at a *p*-value of 0.27.

Binary logistic regression was used to assess associations between dependent and independent variables. Variables with a *p*-value <0.25 were entered into multivariable analysis to determine independent determinants of measles second dose dropout. A significance level of 0.05 was taken as a cutoff value for all statistical significance tests.

## Results

### Sociodemographic characteristics of the study participants

In this study, 446 mothers/caregivers participated, comprising 110 cases and 336 controls, yielding a response rate of 97.8%. The average age of participants was 32.86 years (SD ± 8.2) for cases and 30 years (SD ± 5.4) for controls. The majority of cases, 82 (75.5%) and controls, 229 (68.2%) resided in rural areas. A significant portion of cases, 95 (86.4%) and controls, 327 (97.3%) were married. Moreover, 78 (70.9%) of cases had a family size exceeding five, while 195 (58%) of controls had a family size greater than five (Table 1).

**Table 1:** Sociodemographic characteristics of the study participants in Ejere Woreda, central Ethiopia, 2023 (*N* = 446).

Variables	Category	MCV2 status	
		Cases, *n* (%)	Controls, *n* (%)
Residence	Urban	28 (24.45)	107 (31.84)
	Rural	82 (75.45)	229 (68.15)
Maternal/caregiver age	≤24	22 (20.0)	97 (28.8)
	25–34	41 (37.23)	167 (49.79)
	>35	47 (42.27)	72 (21.42)
Marital status	Married	95 (86.36)	327 (97.32)
	Not married	15 (13.63)	9 (2.67)
Caregivers Educational status	Formal education	61 (55.45)	318 (94.64)
	No-formal education	49 (44.54)	18 (5.34)
Caregivers Occupation	Farmers	82 (74.54)	222 (66.07)
	Merchants	26 (23.63)	94 (27.97)
	Government employee	2 (1.18)	20 (5.95)
Family size	≤5	32 (29.09)	141 (41.96)
	>5	78 (70.9)	195 (58.03)
Birth order	≤3	40 (36.36)	198 (58.9)
	>3	70(63.63)	138(41.07)

### Maternal healthcare-related characteristics

In this study, 52 (47.3%) of the caretakers of cases and 5 (1.5%) of mothers of controls did not receive ANC service during their recent pregnancy. In the study, a very high percentage of cases, 95 (86.6%) and controls, 321 (95.5%) groups delivered at health facilities. Both groups had high postnatal care attendance, with 93 (84.5%) of caretakers of cases and 321 (95.5%) of caretakers of controls attending this service after delivery.

Among the study participants, 52 (47.3%) of the caregivers of cases and 149 (44.3%) of the caregivers of controls traveled for more than 30 min to reach the nearest health facility. Thirty-three (30%) caregivers of cases and 75 (22.3%) caregivers of controls experienced postponement during their vaccination appointment (Table 2).

**Table 2:** Maternal healthcare-related characteristics of study participants in Ejere Woreda, central Ethiopia, 2023 (*N* = 446).

Variables	Categories	MCV2 status	
		Cases, *n* (%)	Controls, *n* (%)
ANC	Yes	58 (52.72)	331 (98.51)
	No	52 (47.27)	5 (1.5)
Number of ANC contact	≤2	57 (51.81)	83 (24.70)
	>2	53 (48.82)	253 (75.29)
Place of delivery	Health facility	95 (86.63)	321 (95.53)
	Home	15 (13.63)	15 (4.47)
Post-natal care attendance	Yes	93 (84.54)	321 (95.53)
	No	17 (15.45)	15 (4.47)
Health care reminder about MCV on PNC	Yes	34 (30.91)	290 (86.3)
	No	76 (69.09)	46 (13.7)
Visited by HEWs in last months	Yes	83 (75.45)	307 (91.37)
	No	27 (24.55)	29 (8.63)
Ever postponed a MCV2 appointment	Yes	32 (29.09)	76 (22.61)
	No	78 (70.90)	260 (77.38)
Health facility vaccination waiting time	Under 30 min	14 (12.73)	119 (35.417)
	Over 30 min	96(87.27)	217(64.583)

### Awareness and perception-related characteristics of the study participants on MCV2

This study found that 79 (71.8%) caregivers of cases and 124 (36.9%) caregivers of controls had no awareness of the second dose of measles (Table 3). Moreover, in nearly three-fourth of cases, 71.8%, and about one-third of the controls, 36.9% had negative perceptions toward the benefits of MCV2.

**Table 3:** Awareness-related characteristics of the study participants in Ejere Woreda, central Ethiopia, 2023 (*N* = 446).

Variable	Category	MCV2 status	
		Cases, *n* (%)	Controls, *n* (%)
Heard about MCV2	Yes	68 (61.81)	31 (9.22)
	No	42 (38.18)	310 (90.77)
Sources of Awareness	Radio	17 (15.45)	52 (15.55)
	Television	13 (11.8)	118 (35.2)
	Health personnel	107 (97.23)	329 (97.9)
	Friends/neighbors	28 (25.25.45)	206 (61.3)
Number of measles vaccine administered	≤1	102 (92.72)	26 (7.73)
	≥2	8 (7.27)	310 (92.26)
Awareness on MCV2	Not aware	79 (71.81)	124 (36.90)
	Aware	31 (28.18)	212(63.09)

### Determinants of measles second dose vaccination dropout

The results of a multivariable logistic regression analysis showed that several variables were significantly associated with dropping out of the second dose of the MCV. These variables include lack of a reminder for the measles vaccine during PNC, lower number of ANC visits, long waiting times for vaccination, lower educational attainment among mothers/caregivers, poor awareness of the second dose of the measles vaccine, and not receiving at least two doses of Vitamin A.

The study found that children's likelihood of missing their second measles vaccine dose was 5.19 times higher (AOR = 5.19; 95% CI: 2.34, 7.83) if their mothers were not reminded during their PNC follow-up. The study also revealed that mothers of children with ≤2 ANC contacts were 4.95 times more likely to have their child drop out of the MCV2, compared to those with >2 ANC contacts (AOR = 4.95, 95% CI: 2.86, 9.24). Similarly, mothers/caregivers who experienced long facility waiting times for vaccination were 2.78 times more likely to have their child drop out of the second measles vaccine dose, compared to those with shorter waiting times (AOR = 2.78, 95% CI: 1.19, 4.38). Children of mothers/caregivers with no formal education were also 6.46 times more likely to drop out of the second dose of the measles-containing vaccine (AOR = 6.46, 95% CI: 2.81, 11.71). Furthermore, mothers/caregivers unaware of the importance of the second dose of measles were 8.37 times more likely to have their child drop out of this dose compared to those who were informed (AOR = 8.37, 95% CI: 4.22, 15.08). Lastly, mothers/caregivers with children not receiving at least two doses of vitamin A were 4.05 times more likely to drop out on the MCV2 (AOR = 4.05; 95% CI: 2.15, 8.11) (Table 4).

**Table 4:** Multivariate analysis of determinants of measles vaccine dropout among 18–24 months old children in Ejere Woreda, central Ethiopia, 2023 (*N* = 446).

Variables	Category	MCV2 status		COR (95% CI)	AOR (95%CI)
		Defaulter, *n* (%)	Complete, *n* (%)		
Residence	Rural	82 (75.45)	229 (68.15)	1.36 (1.02,2.1)	
	Urban	28 (24.45)	107 (31.84)	1	
Maternal/caregiver age	≤24	22 (20.0)	97 (28.8)	0.34 (0.22,0.54)	
	25–34	41 (37.23)	167 (49.79)	0.37 (0.26,0.56)	
	>35	47 (42.27)	72 (21.42)	1	
Marital status	Not married	15 (13.63)	9 (2.67)	5.7 (2.47,10.1)	
	Married	95 (86.36)	327 (97.32)	1	
Caregivers/educational status	No formal education	49 (44.54)	18 (5.34)	14.2 (8.26,22.1)	6.46 (2.81,11.71)*
	Formal education	61 (55.45)	318 (94.64)	1	1
Family size	>5	78 (70.9)	195 (58.03)	1.7 (1.17,2.33)	
	≤	32 (29.09)	141 (41.96)	1	
Birth order	>3	70 (63.63)	138 (41.07)	2.5 (1.67,3.26)	
	≤3	40 (36.36)	198 (58.9)	1	
Antenatal care	Yes	58 (52.72)	331 (98.51)	1	
	No	52 (47.27)	5 (1.5)	59.4 (24.8,94.3)	
Number of ANC contact	≤2	57 (51.81)	83 (24.70)	3.3 (2.27,5.34)	4.95 (2.86,9.24)*
	>2	53 (48.82)	253 (75.29)	1	1
Place of delivery	Home	15 (13.63)	15 (4.47)	3.4 (1.68, 5.57)	
	Health facility	95 (86.63)	321 (95.53)	1	
Post-natal care attendance	No	17 (15.45)	15 (4.47)	3.83 (1.91,6.20)	
	Yes	93 (84.54)	321 (95.53)	1	
Reminder about MCV2 on PNC	No	76 (69.09)	46 (13.7)	14.1 (9.5,20.99)	5.19 (2.34,7.83)*
	Yes	34 (30.91)	290 (86.3)	1	1
Visited by HEWs in last month	No	27 (24.55)	29 (8.63)	3.44 (2.13,5.30)	
	Yes	83 (75.45)	307 (91.37)	1	
Postponement of MCV appointment	Yes	32 (29.09)	76 (22.61)	1.4 (1.02,2.14)	
	No	78 (70.90)	260 (77.38)	1	
Health facility waiting time	Over 30 min	96 (87.27)	217 (64.58)	3.76 (2.46,6.04)	2.78 (1.19,4.38)*
	Under 30 min	14 (12.73)	119 (35.42)	1	1
Awareness on MCV2	Not aware	97 (88.182)	124 (36.9)	12.8 (8.3,20.88)	8.37 (3.44,20.39)*
	Aware	13 (11.818)	212 (63.1)	1	
Received ≥2 doses of Vit-A	No	91 (82.272)	212(63.1)	2.8(1.85,4.13)	4.05 (2.15,8.11)*
	Yes	19(17.27)	124(36.9)	1	1

* Significant at *p*-value < 0.05, 1= Reference.

## Discussion

Understanding the determinants of second-dose measles vaccination dropout is critical for developing effective interventions. This study identified several significant factors contributing to measles second-dose vaccine dropout. Demographic factors such as low educational attainment and unawareness of the second dose, along with health system-related factors including lack of reminders during postnatal care, limited antenatal care visits, lengthy waiting times, and not receiving vitamin A doses, were found to play a crucial role in this issue. Postnatal reminders are crucial in urging mothers to administer the second dose of the measles vaccine, ensuring complete immunization and protection against the disease (22). This study also showed that mothers who weren't reminded about the second measles vaccine dose at their PNC visit were 5.19 times more likely to drop out of it. This aligns with studies conducted in Ethiopia (16, 21) and the Democratic Republic of Congo (23). This might be because mothers might forget the second vaccine dose because they don't receive reminders. They might think skipping the second dose won't affect their child's immunity. It suggests that enhancing communication, improving maternal education, and strengthening policy implementation are necessary steps to ensure complete immunization and protect children against measles.

This study also added that mothers with two or fewer ANC contacts were 4.95 times more likely to drop out of their second measles vaccination. This is supported by studies done in the Afar region of Ethiopia (18, 24, 25), Cameroon (26), Gambia (27), and Zimbabwe (28). This is because fewer ANC visits can create issues for childhood vaccinations. Mothers may miss crucial information about the schedule, have less chance to ask questions, face challenges returning due to scheduling, and be more vulnerable to vaccine misinformation and hesitancy. This highlights the need to strengthen the integration of vaccination education and counseling within ANC contacts.

This study also highlights the significant impact that long waiting times at vaccination sites can have on vaccination dropout rates. The study found that mothers/caregivers who had to wait a long time for measles vaccination were 2.78 times more likely to drop out of the second dose. The studies conducted in the Amhara and Afar regions of Ethiopia (10, 18) support this finding. The reason for this might be that extended waiting times can cause client dissatisfaction, increase vaccination dropout rates, and be inconvenient for caregivers, potentially decreasing motivation to return for subsequent doses.

The study also added that mothers or caregivers without formal education had a 6.46 times higher likelihood of not getting the second dose of the measles vaccine. The finding is in line with the studies conducted in Ethiopia (29, 30), Eritrea (31), and East China (32). It could be related to individuals with lower formal education and limited health literacy may have a misunderstanding of vaccination importance and the risks associated with not completing the vaccine schedule (33).

The study also revealed that mothers/caregivers unaware of the need for a second dose of measles were 8.37 times more likely to drop out of the second dose. This finding is congruent with studies conducted in Ethiopia (10), Kenya (19), and China (32). This might be because, if caregivers weren't properly informed about the importance of the second dose for complete immunity, they might believe their child is protected after receiving the first dose.

Lastly, the study added that children who didn't get at least two doses of vitamin A were 4.05 times more likely to miss their second measles dose. This is in line with the result of the study conducted in Kenya (19). This might be because children who haven't received the recommended vitamin A doses may be from families with lower healthcare-seeking behavior, indicating a pattern of non-compliance with vaccination schedules. This suggests that ensuring access to essential vitamins and educating families on the importance of vaccination could help improve adherence to vaccination schedules.

### Strength and limitations of the study

The study identified a wide range of factors that contribute to the likelihood of children failing to receive their second measles vaccine dose. However, the study relied on self-reported data from mothers/caregivers, and participants were asked to recall vaccination events and related healthcare experiences within the past 12 months, which could introduce recall bias and impact the validity of the findings. Additionally, the lack of matching between cases and controls could have led to confounding bias, obscuring the true associations. The study was also conducted in a specific geographic location, which may limit the generalizability of the findings to other settings. Moreover, including variables with a *p*-value threshold of <0.25 for entry into the multivariable analysis could be the other notable limitation, as it may increase the risk of Type I errors.

## Conclusion

The research uncovered that not receiving reminders during PNC, fewer ANC visits, lengthy waiting times, lack of formal education, lack of awareness, and insufficient vitamin A doses were significant determinants of children aged 18–24 months dropping out of their second measles vaccine dose. Therefore, addressing these key factors through targeted interventions and educational initiatives can improve the uptake of the second measles vaccine among children aged 18–24 months, enhancing vaccination coverage and reducing measles outbreak risk. Moreover, while policies aimed at improving vaccination rates exist, strengthening their implementation is essential for better outcomes. Further implementation research is also needed to assess the fidelity of existing public health interventions related to measles vaccination.

## Data Availability

The original contributions presented in the study are included in the article/Supplementary Material, further inquiries can be directed to the corresponding author.

## References

[1] Panikkath R John K Mascarenhas V (2015). Available online at: https://www.afro.who.int/sites/default/files/2017-06/who-african-regional-measles-and-rubella-surveillance-guidelines_updated-draft-version-april-2015_0.pdf

[2] Organization WH. Measles. (2022). Available online at: https://www.who.int/health-topics/measles#tab=tab_1 (Accessed April 01, 2024).

[3] Organization WH. Measles and rubella strategic framework: 2021-2030; Available online at: https://www.who.int/publications/i/item/measles-and-rubella-strategic-framework-2021-2030 2020 (Accessed April 01, 2024).

[4] Organization WH. Meeting of the Immunization and Vaccine related Implementation Research Advisory Committee (IVIR-AC) September 2022. (2022). Available online at: https://iris.who.int/bitstream/handle/10665/364731/WER9747-eng-fre.pdf?sequence=2 (Accessed April 01, 2024)

[5] Organization WH. Global measles threat continues to grow as another year passes with millions of children unvaccinated. (2023). Available online at: https://www.who.int/news/item/16-11-2023-global-measles-threat-continues-to-grow-as-another-year-passes-with-millions-of-children-unvaccinated (Accessed March 29, 2024).

[6] Organization WH. Measles. (2022). Available online at: https://www.who.int/news-room/fact-sheets/detail/measles (Accessed March 29, 2024).

[7] Indicators K. Mini demographic and health survey. EPHI and ICF. (2019).

[8] Ethiopian Ministry of Health. Health, F.M.o., Expanded Program on Immunization (EPI); Available online at: https://www.moh.gov.et/initiatives-4-col/Expanded_Program_on_Immunization (Accessed March 29, 2024).

[9] Goshu Muluneh A Woldemariam Merid M Tigabu B Getie Ferede M Molla Kassa G Animut Y. Less than one-fifth of Ethiopian children were vaccinated for measles second dose; evidence from the Ethiopian mini demographic and health survey 2019. Vaccine: X. (2022) 12:100217. 10.1016/j.jvacx.2022.100217 36148266 PMC9486014

[10] Tadesse AW Sahlu D Benayew M. Second-dose measles vaccination and associated factors among under-five children in urban areas of North Shoa Zone, Central Ethiopia, 2022. Front Public Health. (2022) 10:1029740. 10.3389/fpubh.2022.1029740 36568740 PMC9780268

[11] Biellik RJ Davis R. The new World Health Organization recommendation on the 2-dose measles vaccine schedule and the way forward in African Region. Pan Afr Med J. (2017) 27(Suppl 3). 29296149 10.11604/pamj.supp.2017.27.3.11611 PMC5745931

[12] Kondamudi NP Waymack JR. Continuing education activity. (2023). Available online at: https://scholar.google.com/scholar?hl=en&as_sdt=0%2C5&q=Kondamudi%2C+N.P.+and+J.R.+Waymack%2C+Continuing+Education+Activity.+2023.&btnG= (Accessed March 29, 2024).

[13] Ullah K Saleem J Zakar R Ishaq M Khattak FA Majeed F Exploring the reasons for defaulting from childhood immunization: a qualitative study in Pakistan. BMC Public Health. (2024) 24(1):408. 10.1186/s12889-024-17926-y 38331754 PMC10851579

[14] Bekele G Darega J Mulu E Tsegaw M. Determinants of immunization defaulters among children aged 12–23 months in Ambo town, Oromia, Ethiopia: a case-control study. Hum Vaccin Immunother. (2024) 20(1):2338952. 10.1080/21645515.2024.2338952 38606820 PMC11018067

[15] Guye AH Nigussie T Tesema M Shambi DB Deriba BS Dureso NS Determinants of defaulter to full vaccination among children aged 12–23 months in Siraro district, West Arsi zone, Oromia, Ethiopia: a case-control study. BMC Pediatr. (2023) 23(1):230. 10.1186/s12887-023-04029-7 37161451 PMC10169456

[16] Chanie MG Ewunetie GE Molla A Muche A. Determinants of vaccination dropout among children 12–23 months age in north Gondar zone, northwest Ethiopia, 2019. Plos One. (2021) 16(2):e0246018. 10.1371/journal.pone.0246018 33556103 PMC7869993

[17] Mekuria DK Hailu G Bedimo M Tefera AA. Determinants of default from full completion of vaccination among children between 12 and 23 months old in Yilmana Densa district, West Gojam zone, Ethiopia, 2019. Front Public Health. (2022) 10:974858. 10.3389/fpubh.2022.974858 36311590 PMC9615568

[18] Hailu C Fisseha G Gebreyesus A. Determinants of measles vaccination dropout among 12− 23 months aged children in pastoralist community of Afar, Ethiopia. BMC Infect Dis. (2022) 22(1):376. 10.1186/s12879-022-07350-1 35421952 PMC9008940

[19] Makokha FM. Uptake of Second Dose of Measles Vaccine Among Children in Kakamega County, Kenya. Juja: Jomo Kenyatta University of Agriculture and Technology (JKUAT) (2017).

[20] Yenit M Assegid S Abrha H. Factors associated with incomplete childhood vaccination among children 12–23 months of age in Machakel Woreda, East Gojjam Zone: aCase-controlStudy. J Pregnancy Child Health. (2015) 2(4):180.

[21] Asfaw AG Koye DN Demssie AF Zeleke EG Gelaw YA. Determinants of default to fully completion of immunization among children aged 12 to 23 months in south Ethiopia: unmatched case-control study. Pan African Medical Journal. (2016) 23(1). 10.11604/pamj.2016.23.100.7879 27222689 PMC4867184

[22] Rasmussen SA Jamieson DJ. What obstetric health care providers need to know about measles and pregnancy. Obstet Gynecol. (2015) 126(1):163–70. 10.1097/AOG.0000000000000903 25899422 PMC4552307

[23] Kayembe-Ntumba H-C Vangola F Ansobi P Kapour G Bokabo E Mandja B-A Vaccination dropout rates among children aged 12–23 months in democratic republic of the Congo: a cross-sectional study. Arch Public Health. (2022) 80(1):18. 10.1186/s13690-021-00782-2 34986887 PMC8728983

[24] Etana B Deressa W. Factors associated with complete immunization coverage in children aged 12–23 months in Ambo Woreda, central Ethiopia. BMC Public Health. (2012) 12:1–9. 10.1186/1471-2458-12-566 22839418 PMC3508824

[25] Girmay A Dadi AF. Full immunization coverage and associated factors among children aged 12–23 months in a hard-to-reach areas of Ethiopia. Int J Pediatr. (2019) 2019. 10.1155/2019/1924941 31263502 PMC6556785

[26] Russo G Miglietta A Pezzotti P Biguioh RM Bouting Mayaka G Sobze MS Vaccine coverage and determinants of incomplete vaccination in children aged 12–23 months in dschang, west region, Cameroon: a cross-sectional survey during a polio outbreak. BMC Public Health. (2015) 15:1–11. 10.1186/s12889-015-2000-2 26156158 PMC4496879

[27] Ntenda PA Sixpence A Mwenyenkulu TE Mmanga K Chirambo AC Andy Bauleni A Determinants of pentavalent and measles vaccination dropouts among children aged 12–23 months in the Gambia. BMC Public Health. (2022) 22(1):520. 10.1186/s12889-022-12914-6 35296298 PMC8926885

[28] Mukungwa T. Factors associated with full immunization coverage amongst children aged 12–23 months in Zimbabwe. African Population Studies. (2015) 29(2). 10.11564/29-2-745

[29] Kinfe Y Gebre H Bekele A. Factors associated with full immunization of children 12–23 months of age in Ethiopia: a multilevel analysis using 2016 Ethiopia demographic and health survey. PloS One. (2019) 14(11):e0225639. 10.1371/journal.pone.0225639 31774859 PMC6881024

[30] Nour TY Farah AM Ali OM Osman MO Aden MA Abate KH. Predictors of immunization coverage among 12–23 month old children in Ethiopia: systematic review and meta-analysis. BMC Public Health. (2020) 20:1–19. 10.1186/s12889-019-7969-5 33243208 PMC7689978

[31] Kibreab F Lewycka S Tewelde A. Impact of mother’s education on full immunization of children aged 12–23 months in Eritrea: population and health survey 2010 data analysis. BMC Public Health. (2020) 20(1):267. 10.1186/s12889-020-8281-0 32087706 PMC7036221

[32] Hu Y Li Q Luo S Lou L Qi X Xie S. Timeliness vaccination of measles containing vaccine and barriers to vaccination among migrant children in east China. PloS One. (2013) 8(8):e73264. 10.1371/journal.pone.0073264 24013709 PMC3755000

[33] Novilla MLB Goates MC Redelfs AH Quenzer M Novilla LKB Leffler T Why parents say No to having their children vaccinated against measles: a systematic review of the social determinants of parental perceptions on MMR vaccine hesitancy. Vaccines (Basel). (2023) 11(5):926. 10.3390/vaccines11050926 37243030 PMC10224336

