# Supplementary material for: Determinants of measles second dose vaccination dropout among children aged 18–24 months in Ejere woreda, central Ethiopia; unmatched case-control study
Source: Front Pediatr. 2024 Sep 18;12:1432762. doi: 10.3389/fped.2024.1432762 (PMC11445012; doi:10.3389/fped.2024.1432762)
Supplement: Supplementary file 1 [file Table1.pdf]

Supplementary Table 1

	**Part I: Socio-demographic characteristics**	CODE: _________	
S/No.	Question	Response Categories	Skip to
101	Place of residence	1. Urban<br>2. Rural	
102	Who is the caregiver of a child?	1. Mother<br>2. Father<br>3. Grandparent<br>4. Older sibling<br>5. Other(specify)_____________	
103	Mother/Caregiver's age	____________________	
104	Marital status?	1. Married<br>2. Separated<br>3. Divorced<br>4. Widowed	
105	Educational status?	1. No formal education<br>2. Primary school<br>3. Secondary school<br>4 College and above	
106	Occupation?	1. Farmer<br>2. Merchant<br>3. Government employee<br>4. Daily laborer<br>5. Housewife<br>6. Other(specify)	
107	Family size in the household in no.?	_______________	
108	A total number of ever born by mother?	________________	
109	Total No of children alive?	________________	
110	Sex of the child?	1. Male<br>2. Female	
111	Age of the child in a month?	__________________	
112	Birth order?	__________________	
113	What is the preceding birth interval?	______________________	
**Part. II Maternal Health Care Related factors**			
201	Did the mother have attended antenatal care during the last pregnancy?	1. Yes<br>2. No	No, Skip to Q. 203
202	If yes, how many times did she attend antenatal care during her pregnancy?	________________	
203	Where did the mother deliver her baby?	1. Home<br>2. Health center	

		3. Health post<br>4. Hospital	
204	Have attended Post Natal Care service after the delivery of her child?	1. Yes<br>2. No	No, Skip to Q.206
205	If yes, how many times did she attend the Post Natal Care service after the delivery of your child?	____________________	
206	Was a healthcare reminder given to the mother on the measles vaccination?	1. Yes<br>2. No	
**Part. III Health facility-related factors**			
301	How long it takes you to reach the nearest vaccination site? (in minutes)	____________________	
302	Have you been visited by HEWs at your home in the last month?	1. Yes<br>2. No	No skip to Q.304
303	How many times you have been visited by HEWs at your home in the last month?	__________________	
304	Have you ever postponed measles vaccine appointment schedule of your child?	1. Yes<br>2. No	No, Skip to Q.306
305	What was the reason? (More than one answer possible)	1. Fear of side effects<br>2. Mother’s too busy<br>3. Vaccinators were Absent<br>4. Absence of vaccination<br>5. Forgetting the day of vaccination<br>6. Child sickness<br>7. Other(specify)	
306	How many days are used for measles vaccination weekly?	1. Two<br>2. Three<br>3. Five<br>4. Seven	
307	How long is the weighting time to get the measles vaccine in the facility?	1. <30 min<br>2. 30min-1hr<br>3. 1-2hrs<br>4. >2hrs	

	**Part IV. Awareness about the second dose of Measles**		
401	Have you heard about the second dose of the measles vaccination?	1. Yes<br>2. No	No skip to Q.404
402	Your source of hearing about measles vaccination?	1. Radio<br>2. TV<br>3. Health personnel<br>4. Friends/Neighbors<br>5. Other(specify)	
403	How many times measles vaccine Should be administered?	1. One<br>2. Two<br>3. Four<br>4. I didn't know	
404	Age at first administration of measles?	1. 9month<br>2. 12month<br>3. 18month<br>4. I didn't know	
405	Age at the administration of the second dose of measles?	1. After 4 weeks first MCV<br>2. After 2month the first MCV<br>3. After 5month the first MCV<br>4. I didn't know	
406	How many times do children visit the immunization site to be fully protected?	1. One<br>2. two<br>3. Five<br>4. Six<br>5. I didn't know	
	**Part V: Perception toward the benefit of MCV2 vaccination**		
501	A child growing well so no need for MCV2 Vaccination?	1. Strongly disagree<br>2. Disagree<br>3. Not sure<br>4. Agee<br>5. strongly agree	
502	Measles immunizations provide lifelong Protection?	1. Strongly disagree<br>2. Disagree<br>3. Not sure<br>4. Agee	

		5. Strongly agree	
503	Measles immunizations are harmful to children?	1. Strongly disagree<br>2. Disagree<br>3. Not sure<br>4. Agree<br>5. Strongly agree	
504	Measles immunizations prevent Life-threatening diseases?	1. Strongly disagree<br>2. Disagree<br>3. Not sure<br>4. Agee<br>5. Strongly agree	
505	All children are susceptible to Vaccine-preventable measles diseases?	1. Strongly disagree<br>2. Disagree<br>3. Not sure<br>4. Agee<br>5. Strongly agree	
506	Adherence to and completion of measles immunization Schedules are important for child development.	1. Strongly disagree<br>2. Disagree<br>3. Not sure<br>4. Agee<br>5. Strongly agree	
	**Part VI: Immunization related to measles**		
601	Do you have your child's Immunization card?	1. Yes<br>2. No	No, skip to 603
602	If yes, check from card	1. BCG<br>2. PENTA3<br>3. Vitamin A at 6 Months<br>4. Vitamin A at 12 Months<br>5. Vitamin A at 18 Months<br>6. Vitamin A at 24 Months	
603	If No, ask for history orally	1. BCG<br>2. PENTA3<br>3. Vitamin A at 6 Months<br>4. Vitamin A at 12 Months<br>5. Vitamin A at 18 Month<br>6. Vitamin A at 24 Months	

1. Organization WH. Global measles threat continues to grow as another year passes with millions of children unvaccinated. 2023.
